# Climate Change and Coffee Quality: Systematic Review on the Effects of Environmental and Management Variation on Secondary Metabolites and Sensory Attributes of *Coffea arabica* and *Coffea canephora*

**DOI:** 10.3389/fpls.2021.708013

**Published:** 2021-10-08

**Authors:** Selena Ahmed, Sarah Brinkley, Erin Smith, Ariella Sela, Mitchell Theisen, Cyrena Thibodeau, Teresa Warne, Evan Anderson, Natalie Van Dusen, Peter Giuliano, Kim Elena Ionescu, Sean B. Cash

**Affiliations:** ^1^Food and Health Lab, Department of Health and Human Development, Montana State University, Bozeman, MT, United States; ^2^Department of Horticultural Sciences, Center for Coffee Research and Education, Texas A&M University, College Station, TX, United States; ^3^Friedman School of Nutrition Science and Policy, Tufts University, Boston, MA, United States; ^4^Essy's Coffee, Bozeman, MT, United States; ^5^Treeline Coffee Roasters, Bozeman, MT, United States; ^6^Specialty Coffee Association, Santa Ana, CA, United States; ^7^Coffee Science Foundation, Santa Ana, CA, United States

**Keywords:** crop quality, secondary metabolites, sensory attributes, altitude, agricultural systems, climate change, coffee plants

## Abstract

Climate change is impacting crop performance and agricultural systems around the world with implications for farmers and consumers. We carried out a systematic review to synthesize evidence regarding the effects of environmental factors associated with climate change and management conditions associated with climate adaptation on the crop quality of a culturally-relevant perennial crop, coffee (*Coffea arabica* and *Coffea canephora*). Seventy-three articles were identified that addressed the study's research question including 42 articles on environmental factors, 20 articles on management conditions, and 11 articles on both. While variation was found between studies, findings highlight that coffee quality is vulnerable to changes in light exposure, altitude, water stress, temperature, carbon dioxide, and nutrient management. Both increases as well as decreases were found in secondary metabolites and sensory attributes that determine coffee quality in response to shifts in environmental and management conditions. The most consistent evidence identified through this systematic review includes the following two trends: (1) increased altitude is associated with improved sensory attributes of coffee and; (2) increased light exposure is associated with decreased sensory attributes of coffee. Research gaps were found regarding the effects of shifts in carbon dioxide, water stress, and temperature on the directionality (increase, decrease, or non-linear) of coffee quality and how this varies with location, elevation, and management conditions. This systematic review further identified the following research needs: (1) long-term studies that examine the interactive effects of multiple environmental factors and management conditions on coffee quality; (2) studies that examine the interaction between sensory attributes and secondary metabolites that determine coffee quality and; (3) studies on the feasibility of various climate-adaptation strategies for mitigating the effects of climate change on coffee quality. Evidence-based innovations are needed to mitigate climate impacts on coffee quality toward enhanced sustainability and resilience of the coffee sector from farm to cup.

## Introduction

Climate change is impacting agricultural systems around the world with implications throughout the food system and for society broadly. The past seven decades have experienced a rise in atmospheric carbon dioxide (CO_2_) concentrations that correspond with gradual systematic changes in average climate conditions including increased variability of temperature and precipitation as well as more frequent extreme weather conditions (Pachauri et al., 2014). These climate-driven changes are impacting crops in multiple ways including changes in: (1) the geographic ranges suitable for cultivation (Porter and Semenov, 2005); (2) trophic interactions in agroecosystems (Ahmed et al., 2014a); (3) crop productivity (Lobell and Asner, 2003) and; (4) crop quality (determined based on concentrations of micronutrients, minerals, and secondary metabolites; Myers et al., 2014; Ahmed and Stepp, 2016; Ahmed et al., 2019). Climate-driven changes in agricultural systems have notable implications for farmer livelihoods and management decisions as well as for consumer experiences and wellbeing (DaMatta et al., 2010; Ahmed et al., 2014b; Ahmed and Stepp, 2016; Boehm et al., 2019). Given projected increased mean temperatures and atmospheric CO_2_ levels as well as more variable precipitation regimes (Pachauri et al., 2014), it is essential to understand how climate change is impacting different crops in various management systems in order to design evidence-based strategies to strengthen the resilience of agriculture to global change (Han et al., 2020).

Studies over the past few decades provide notable evidence on the effects of climate change on crop yields and shifts in crop geographic ranges (Porter and Semenov, 2005). However, fewer studies have focused on effects on crop quality (Myers et al., 2014; Ahmed and Stepp, 2016). Crop quality is a multi-dimensional outcome that is driven by the presence, absence, or change in concentrations of primary metabolites (i.e., sugars, lipids, vitamins, and minerals) and secondary metabolites (also knowns as phytochemicals) (Mattos et al., 2014; Ahmed and Stepp, 2016). The presence and concentration of primary and secondary metabolites in crops influences sensory attributes (i.e., color, visual appearance, aroma, taste, and texture), shelf life, bioactivity, and nutritional and health properties (Mattos et al., 2014; Ahmed and Stepp, 2016).

Previous research highlights the vulnerability of secondary metabolites and crop quality to climate change (Ahmed et al., 2019). For example, increased atmospheric CO_2_ drives the CO_2_ fertilization effect of increased crop growth which is associated with a dilution effect for crop quality. Specifically, the CO_2_ fertilization effect results from increased atmospheric CO_2_ that increases net photosynthesis rates in plants as well decreases stomatal conductance and ultimately increases crop yield (Ainsworth and Rogers, 2007). The increase in crop growth with the CO_2_ fertilization effect is associated with the dilution effect in plants of decreased metabolite concentrations (Jarrell and Beverly, 1981). For instance, evidence highlights reduced concentrations of zinc (Zn), iron (Fe), and protein in C_3_ grains grown under elevated conditions of CO_2_ (Myers et al., 2014). In addition to changes due to CO_2_, previous studies have highlighted that changes in water stress, light exposure, and temperature can impact concentrations of secondary metabolites of various crops (Ahmed and Stepp, 2016) including tea plants (Ahmed et al., 2019). Environmental-driven changes in crop quality may be offset by climate adaptation strategies and other management conditions (Ahmed et al., 2014a).

However, there remain knowledge gaps regarding the impacts of climate change on crop quality for numerous plant species which limits the ability for climate adaptation. Given current and predicted climate change trends, climate adaptation is called for to strengthen the sustainability and resilience of the coffee system. In contrast to annual crops, perennial crops such as coffee require longer time periods for farmers and other stakeholders to make decisions. Research is thus needed to synthesize evidence regarding both the effects of environmental factors related to climate change as well as management conditions related to climate adaptation on coffee quality toward informing climate mitigation and adaptation in the coffee system. Examining the effects of environmental and management conditions associated with climate change is especially important given the lack of studies directly examining the long-term effects of climate change on coffee quality. This systematic review synthesizes the totality of published evidence on the following research question: *What are the effects of environmental factors related to climate change and management conditions linked to climate adaptation on coffee quality on the basis of secondary metabolites and sensory attributes?* Coffee was selected as a study system because it is a culturally-relevant perennial beverage crop that is vulnerable to climate change (Bunn et al., 2015; Ovalle-Rivera et al., 2015; Moat et al., 2017; DaMatta et al., 2019; Solymosi and Techel, 2019).

This systematic review is intended to contribute to the scientific literature on the effects of climate change on crop quality. Specifically, this systematic review contributes to the scientific literature through the identification of the following:

1. Identification of the environmental and management factors where the greatest evidence exists with regards to the number of published articles and the consistency of evidence across studies regarding effects on coffee quality.
2. Identification of the environmental and management factors where evidence gaps exist with regards to the number of published articles and the lack of consistency of evidence across studies regarding effects on coffee quality.
3. Identification of the coffee quality parameters where the greatest and least evidence exists with regards to the number of published articles.
4. Identification of research questions and topics for future research toward designing evidence-based innovations for enhancing the resilience of the coffee system to global change.

## Background

Coffee, sourced from the *Coffea arabica* (Arabica coffee) and *Coffea canephora* (Robusta coffee) plants, is among the most widely consumed beverage crops globally (ICO, 2020). Native to Ethiopia, coffee is cultivated in ~80 tropical and subtropical countries (FAOSTAT, 2018). Over 11 million ha are dedicated to coffee production worldwide (FAOSTAT, 2018). Coffee is cultivated across an estimated 12.5 million coffee farms that are largely managed by smallholder farmers (Enveritas, 2019) that own <2 ha (Lowder et al., 2016). Coffee production and trade notably contributes to the livelihoods and economies in the areas where it is produced (Gresser and Tickell, 2002; Waller et al., 2007; ICO, 2020).

Coffee production is sensitive to variation in temperature and precipitation outside the range of its' optimal growing conditions. The optimal growing conditions for Arabica coffee plants are temperatures between 14 and 26°C, annual rainfall between 1,000 and 2,700 mm, and a dry period of 1–3 months annually (Clifford and Willson, 1985; Davis et al., 2006; Wakjira, 2006; Ovalle-Rivera et al., 2015). Coffee's optimal growing conditions are typically found at altitudes of ~400–1,200 meters above sea level (m.a.s.l.) in tropical regions with latitudes 9–27°N/S, or altitudes of ~1,000–2,100 m.a.s.l. in equatorial regions <9°N/S (Illy and Viani, 2005; Ovalle-Rivera et al., 2015).

Coffee-producing regions are increasingly experiencing climate conditions outside optimal ranges (Vinecky et al., 2017) including heat waves and droughts that are expected to impact coffee production and its geographic range (Davis et al., 2006; Bunn et al., 2015; Vinecky et al., 2017). For example, the coffee-producing region of Central America is recognized to be a climate change hotspot of vulnerability and risk with increased occurrence of droughts, hurricanes, and the El Niño-southern oscillation (ENSO) phenomena (Giorgi, 2006). Climate change scenarios for Central America indicate a drastic reduction in the area suitable for coffee by 2050 (Läderach et al., 2017). For instance, over 90% of the coffee-growing area in Nicaragua is projected to experience a decrease in suitability of growing conditions for coffee production in the next 30 years, with farms located at lower altitudes being more vulnerable (Läderach et al., 2017). In contrast, several regions in East Africa and Asia have been identified where climate change is expected to increase the suitability of growing conditions for coffee production (Bunn et al., 2015). However, coffee cultivation in new opportunity regions may require notable land-use changes such as deforestation that is associated with loss of biodiversity and increased greenhouse gas emissions.

Along with shifts in coffee yields and areas suitable for production, coffee quality is also vulnerable to climate change (Läderach et al., 2017). Coffee quality is determined by the presence and concentrations of primary and secondary metabolites that influence sensory (i.e., organoleptic) attributes, shelf stability, and nutritional aspects of coffee (Flament and Bessière-Thomas, 2002; Folmer, 2017). In the specialty coffee industry, high-quality coffee is often defined by a “balanced” cup that is characterized by specific levels of acidity and body as well as flavor (aroma and taste) and aftertaste attributes (Lingle, 1986; Folmer, 2017). The sensory profile of a “balanced” cup of coffee is linked to its' secondary metabolite composition including concentrations of caffeine (a methylxanthine alkaloid), trigonelline (an alkaloid), various chlorogenic acids (polyphenolic compounds), and a range of volatile compounds (terpenoids) (Frank et al., 2007; Hii and Borém, 2019). Coffee aroma is a key sensory attribute of coffee quality that is derived from hundreds of volatile secondary metabolites (including alcohols, aldehydes, hydrocarbons, and ketones) that account for <0.2% of the weight of roasted coffee (Viani, 2000). Approximately 20–25 compounds of coffee's volatile profile dictate its' overall aroma perception, with prevalent compounds being 2-ethyl-3,5-dimethylpyrazine, methylpropanal, 3-methylbutanal, methanethiol, 2- furfurylthiol, 3-mercapto-3-methylbutyl formate, and (*E*)-ß-damascenone (Buffo and Cardelli-Freire, 2004; Dunkel et al., 2014). In addition, several types of amino acids impart a sweet taste to coffee (Worku et al., 2018). Amino acids further serve as precursors to other flavor compounds in coffee (Worku et al., 2018).

Coffee quality varies at each step of production from farm to cup. On farm, coffee quality is influenced by genetics (Dessalegn et al., 2007), climate and other environmental factors (Bertrand et al., 2012), management conditions (Vaast et al., 2006), and harvest (Taveira et al., 2014). Post-harvest, coffee quality is influenced by processing, storage, and preparation (Viani, 2000; Schwan and Fleet, 2015). For example, coffee production in low altitude regions in Nicaragua are vulnerable to reduced acidity and flavor due to climate change (Läderach et al., 2017). In areas of Ethiopia experiencing low and erratic rain, supplemental irrigation in coffee production systems was found to improve coffee quality on the basis of acidity, body, and overall flavor (Tesfaye et al., 2013). In Costa Rica, management conditions including fruit thinning and fungicide application to combat coffee leaf rust altered volatile secondary metabolite composition and the perceived quality of the prepared coffee beverage (Echeverria-Beirute et al., 2018). Ultimately, the sensory and secondary metabolite profiles that determine coffee quality influence consumer purchasing decisions, with economic implications throughout the coffee system.

## Methods

This systematic review follows the methodology of a recent systematic review on climate effects on tea quality (Ahmed et al., 2019). Due to the lack of studies examining the long-term effects of climate change on coffee quality, we examined the effects of multiple environmental factors associated with climate change as well as management conditions associated with climate adaptation. The systematic review protocol for this study was designed by a multisectoral and multidisciplinary team of subject experts from academia and the coffee industry including those with expertise in agricultural economics, climate change, coffee sourcing and sales, coffee science, food systems, phytochemistry, plant biology, sensory science, soil science, and sustainability.

We used the Preferred Reporting Items for Systematic Reviews and Meta-Analyses (PRISMA; Moher et al., 2009) and the PICO (Population, Intervention/Exposure, Comparator, Outcome) framework elements (Schardt et al., 2007) to design the systematic review protocol to collect evidence on the following two closed-frame study questions: (1) *What are the effects of environmental factors related to climate change on coffee quality;* and (2) *What are the effects of management conditions related to climate change adaptation on coffee quality?*

The PICO framework included the following elements: (1) biological population/study unit: coffee (*Coffea* spp.*;* Rubiaceae; *Coffea arabica* and *Coffea canephora*); (2) intervention/exposures: environmental factors linked to climate change and management conditions linked to climate change adaptation (including altitude, geography, light exposure, precipitation, temperature, herbivory, disease, and soil); (3) comparators: variation in specific environmental and management conditions from a baseline point of evaluation and; (4) outcome: coffee quality. Specifically, the outcome of coffee quality was evaluated based on: (1) primary metabolites (sugars, lipids, and minerals); (2) secondary metabolites (polyphenols, alkaloids, terpenoids/volatiles, and amino acids) and; (3) sensory attributes (taste, aroma, mouthfeel, and body).

The development of the systematic review protocol started with scoping search terms in order to test the search strategy for the volume of relevant literature. The final set of search terms was identified through an iterative process after examining relevant articles and refining search terms. The Boolean phrasing utilized to identify relevant articles in the databases consisted of iterations of the following search terms: [iterations of coffee] AND [climate change OR iterations of environmental OR iterations of management] AND [quality OR iterations of secondary metabolites OR iterations of sensory attributes]. See Supplementary Tables 1, 2 for the final search terms.

The final search was conducted using five databases that were selected due to their use in the fields of agriculture, climate science, food systems, food science, plant sciences, and phytochemistry including: Web of Science, EBSCO GreenFILE, Agricola, PubMed, and ProQuest. The search condition criteria included all peer-review articles published in the English language from 2000 to September 2018. The search resulted in the retrieval of 2,641 articles including 949 duplicates, for a total of 1,692 unique articles.

The 1,692 retrieved articles were imported to the Covidence software program, an online platform that facilitates collaborative article screening, reviewing, and data extraction for systematic reviews. Members of a panel of eight reviewers determined if the articles met the *a priori* inclusion criteria of the systematic review protocol. Two reviewers of the review panel were randomly selected by the Covidence software program to screen each article title and abstract to minimize bias in identifying articles to include in the systematic review based on the following *a priori* inclusion criteria: (1) the title or abstract addresses at least one of the close-framed research questions driving the systematic review; (2) the abstract reports on at least one coffee quality parameter (metabolites or sensory attributes).

**Figure 1:**
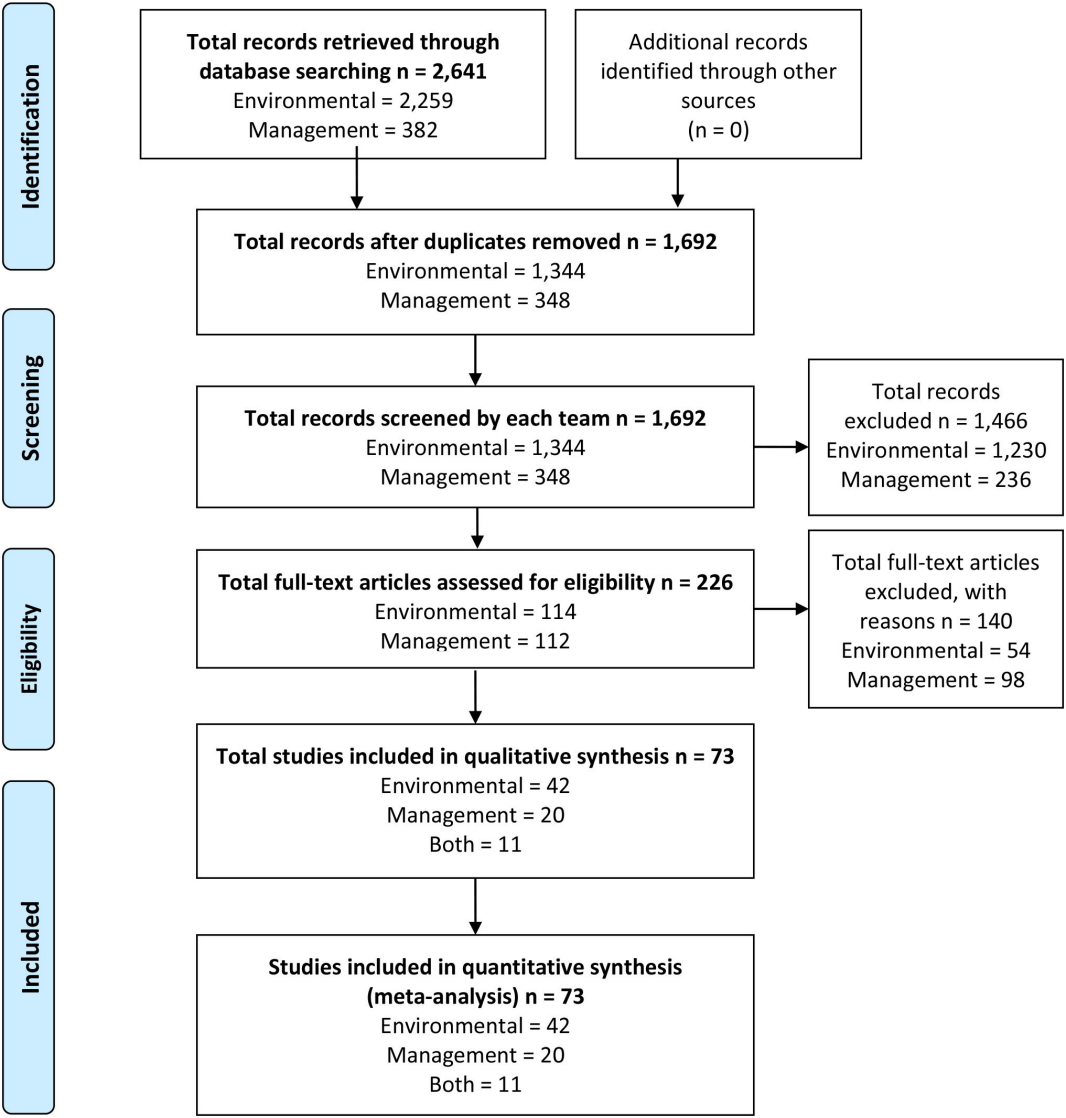
PRISMA Flow Diagram detailing the systematic literature review process for articles assessing the effects of environmental and management conditions on coffee quality from 2000 to 2018. The database search resulted in a final 73 eligible articles for qualitative analysis including 42 articles focused on environmental factors, 20 articles focused on management conditions, and 11 articles focused on both environmental and management conditions.

Articles that met the inclusion criteria for the title and abstract screening process were then read in their entirety by two members of the review panel to determine eligibility to be included in the systematic review. Articles that met the following *a priori* criteria during the full-text eligibility screening were included in the systematic review: (1) the article presents original primary research (not a review or analysis of secondary data); (2) is peer-reviewed; (3) is published in the English language; (4) was published between 2000 to 2018 and; (5) the full text of the article is available. Discrepancies between the raters with regards to inclusion during the screening process were discussed by a minimum of three panelists on the research team for resolution. The review panel identified 59 articles that met the *a priori* inclusion criteria for the systematic review for the closed-frame question on environmental factors and identified 14 articles that met the *a priori* inclusion criteria for the closed-frame question on management conditions (Figure 1 depicts the PRISMA Flow Diagram).

The articles included in the systematic review were read, critically appraised, and extracted for the following data: (1) location where the study was conducted; (2) variety/cultivar of coffee; (3) environmental factor(s) and/or management condition(s); (4) coffee quality parameter(s) and; (5) summary of study outcome(s). The extracted data were reviewed by a second member on the review panel for verification and validity. Articles included in the systematic review were then grouped on the basis of the environmental factor(s) and/or management condition(s) which each study focused on. The environmental factors and management conditions assessed include: altitude (elevation, slope aspect, position), geographic location (geographic origin, geography, and seasonality), light exposure (light intensity, solar radiation, plant density, shade, shade trees), cultivar, nutrient management (soil micronutrient, fertilization, soil composition and chemical characteristics), pests and disease, water stress (precipitation, irrigation, drought, moisture stress water availability), temperature, and carbon dioxide. Articles investigating multiple environmental factors and/or management conditions were assigned to two or more groups. Given that some articles reported on multiple environmental factors and/or management conditions, the sum of the number of studies reported for each environmental factor and management condition is greater than the total number of articles included in this systematic review.

A coding framework adapted from the systematic review methodology of Ahmed et al. (2019) on tea quality was applied to quantify the number of articles where the outcome of coffee quality demonstrated a specific response to variation in environmental factors and/or management condition(s). Specifically, articles were coded with a 0 for absence or a 1 for presence for each of the following coffee quality outcomes: increase, decrease, or no change in [sensory attributes OR secondary metabolites] with variation (increase OR decrease) in a specific environmental factor or management condition. See Supplementary Table 3 for categorization of coffee quality outcomes. Two reviewers coded the outcomes for each article and the review panel resolved discrepancies. Since studies had variable experimental designs and approaches for measuring coffee quality, further quantitative comparison of the amount of change of a quality parameter was not possible between studies.

## Results

### Identifying Environmental and Management Factors in Articles

The systematic review process identified 73 articles that addressed the inclusion criteria including 42 articles that focused on environmental factors associated with climate change, 20 articles focused on management conditions associated with climate change adaptation, and 11 articles focused on both. Ten prevalent environmental factors and management conditions were identified in the studies including: (1) geography; (2) altitude; (3) light exposure; (4) temperature; (5) water stress; (6) nutrient management; (7) type of cultivar; (8) pests and disease management; (9) fruit thinning and; (10) carbon dioxide (Table 1). The findings below are presented on the basis of these variables.

**Table 1:** Number of articles for each environmental factor and management condition.

**Number of articles**	**Environmental factor or management condition**	**Sub-factors**	**Corresponding figure number**
31	Geography	Geographic origin, climate/seasonality, latitude/longitude, topography	Figure 2
18	Altitude	Elevation above sea level, range of altitudes	Figure 3
19	Light exposure	Light intensity, shade intensity, plant density, shade tree diversity/distribution, sunlight, solar radiation, sun-exposed, shade, slope aspect	Figure 4
9	Temperature	Average daily temperature, low temperature, min/max temperature, temperature differences, potential evaporation	Figure 5
9	Water stress	Irrigation, precipitation, annual precipitation, rainfall, moisture stress, water availability, drought	Figure 6
8	Nutrient management	Micronutrients, nitrogen fertilization, ratio of nutrients, soil acidity, base saturation, soil chemical characteristics, form of fertilization	Figure 7
3	Type of cultivar	Cultivar, disease resistant cultivar	Na
2	Pests and disease	Coffee berry borer, pests, disease	Na
1	Fruit thinning	Manual fruit thinning	Na
1	Carbon dioxide	Elevated CO_2_ concentration	Na

**Figure 2:**
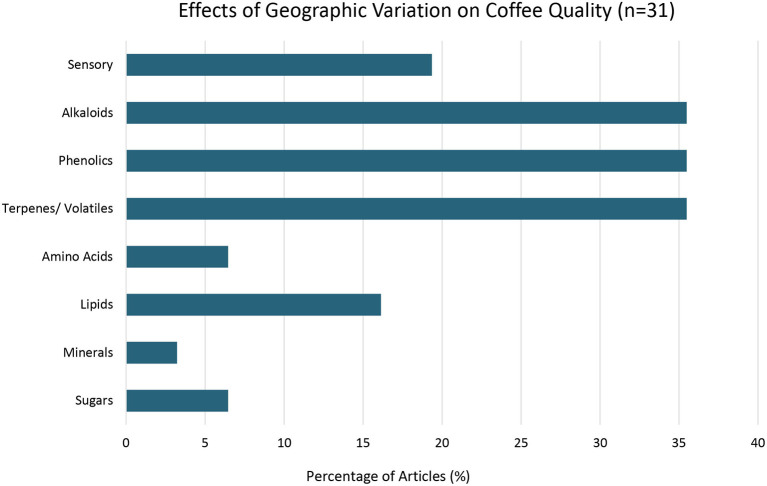
Effects of Geographic Variation on Coffee Quality. Percent of articles out of a total of 31 articles examining geography that found shifts in specific coffee quality parameters with geographic variation.

For the outcome of coffee quality, 26 articles measured the presence and/or concentrations of secondary metabolites in green or roasted coffee beans and/or brewed coffee; 18 articles measured sensory attributes of green or roasted coffee beans and/or brewed coffee (*n* = 18) and; 5 articles measured primary metabolites in coffee. Twenty-three articles measured multiple coffee quality parameters. Prevalent secondary metabolites measured in the articles include caffeine, chlorogenic acids (3-, 4-, and 5-*O-*caffeoylquinic acid), diterpenes (kahweol and cafestol), and other volatiles. The main primary metabolites measured in the reviewed studies are lipids/fatty acids, sugars, proteins, and amino acids. Prevalent sensory attributes measured in the studies include acidity, body, aroma/fragrance, taste, and balance.

The articles included in this systematic review were conducted in a range of coffee-producing regions with the majority carried out in Brazil (*n* = 26) followed by Ethiopia (*n* = 13). Studies were also carried out in Colombia (*n* = 7), Kenya (*n* = 4), India (*n* = 4), and Costa Rica (*n* = 4), among other countries. Fifty-one articles included *Coffea arabica* (Arabica coffee) as the study system, 11 articles included both *Coffea arabica* and *Coffea canephora* (Robusta coffee), and five articles included only *Coffea canephora* as the study system. Three articles included one or more species in addition to *Coffea arabica* and *Coffea canephora* as the study system (Dussert et al., 2001, 2008; Rubayiza and Meurens, 2005) while three other articles did not specify the species (Hamon et al., 2017; Hagos et al., 2018; Walker et al., 2018). Two articles assessed wild *Coffea* species as the study system (Campa et al., 2005; dos Santos Scholz et al., 2016). Multiple coffee varieties and cultivars were examined in several of the reviewed articles while other articles did not specify this information. Given the lack of studies specifying the coffee variety and cultivar, we were unable to compare how environmental and management conditions impact coffee plants based on variety and cultivar.

The following sections detail how specific environmental and management conditions impact coffee quality. Examples are provided of the interactive effects of multiple environmental factors and/or management conditions.

### Geography

Geography was included as an environmental factor in this study as the geographic areas suitable for agriculture are shifting with climate change (Ahmed et al., 2019). This systematic review found that cultivating coffee in different geographic areas was associated with variation of key secondary metabolites driving coffee quality including variation in concentrations of methylxanthines/alkaloids (11 articles), phenolics (11 articles), and terpenes/volatiles (11 articles). For example, two articles found that geographic variation resulted in shifts in important odor-active markers for distinguishing geographic origin of coffee including the volatiles 2,3-butanedione, 2,3-pentanedione, 2-methylbutanal, and 2,3-dimethylpyrazine (de Toledo et al., 2017; Dryahina et al., 2018).

Several articles demonstrated that cultivating coffee in different geographic areas resulted in changes in multiple primary metabolites including: (1) lipids (Decazy et al., 2003; Dussert et al., 2008; Dong et al., 2015; dos Santos Scholz et al., 2016; Sant'Ana et al., 2018); (2) sugars (Avelino et al., 2005; dos Santos Scholz et al., 2016) and; (3) amino acids (Dong et al., 2015; dos Santos Scholz et al., 2016), and minerals (Muñiz-Valencia et al., 2013). Six articles in this review found that cultivating coffee in different geographic areas was associated with variation in sensory attributes (Avelino et al., 2005; Läderach et al., 2011; Oberthür et al., 2011; Kitzberger et al., 2016; Yener et al., 2016; Malau et al., 2017). For example, when *Coffea arabica* is transplanted outside of its place of origin, Ethiopia, lipid content decreased while both lipid and diterpene content show increased variation (Sant'Ana et al., 2018).

Several studies highlight the interaction of geography with genetic diversity of coffee germplasm. For example, coffee quality parameters demonstrated variation within a narrow geographic range for wild Ethiopian Arabica accessions that are recognized to have large genetic diversity (dos Santos Scholz et al., 2016). Alternatively, cultivars that have less genetic diversity demonstrated less variation in coffee quality based on wide geographic distribution (Avelino et al., 2005; Sant'Ana et al., 2018).

### Altitude

**Figure 3:**
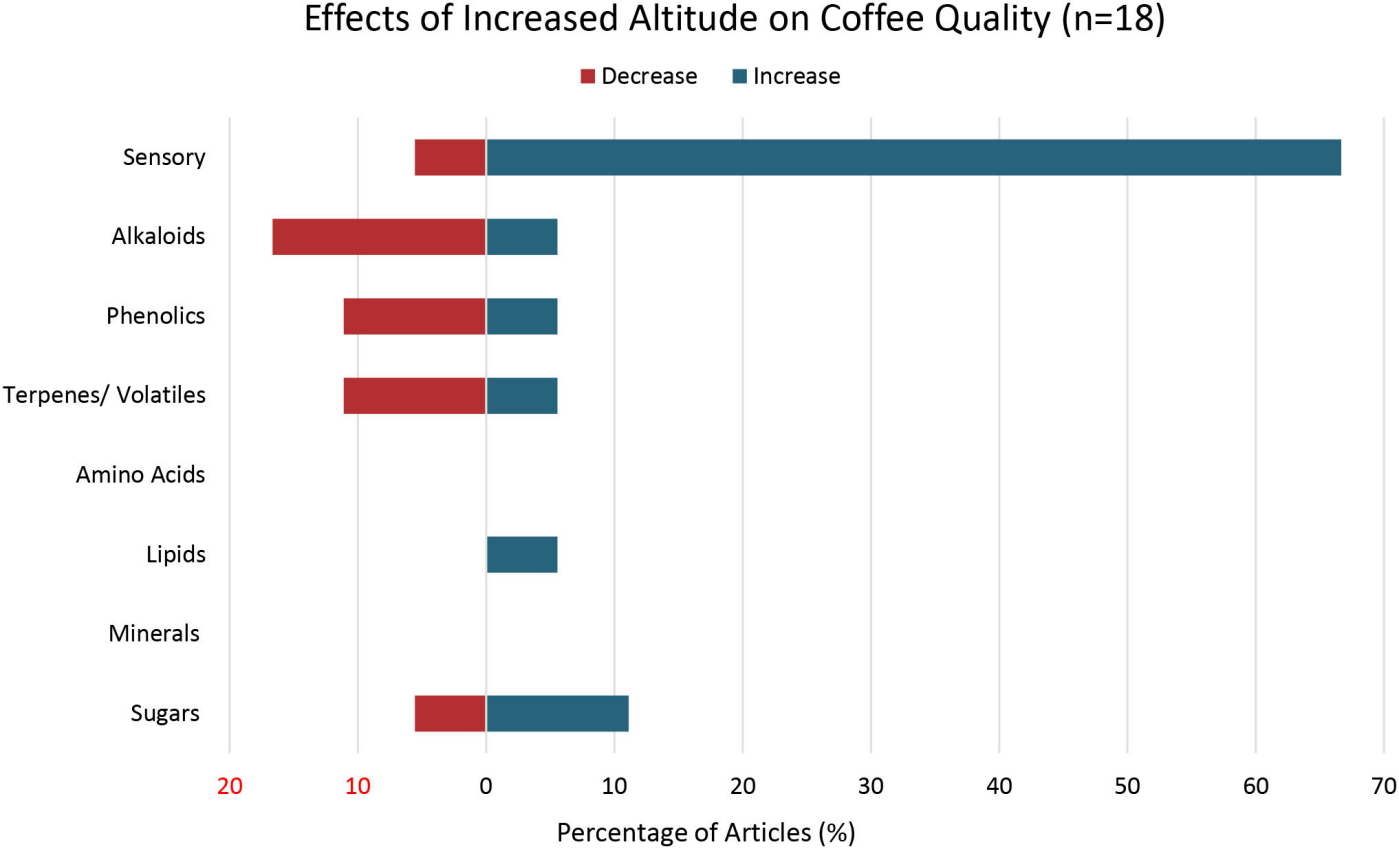
Effects of Increased Altitude on Coffee Quality. Percent of articles out a total of 18 articles that examined altitude that reported either an increase, decrease, or both increase and decrease in specific coffee quality parameters with increased altitude.

Altitude was included as an environmental factor in this systematic review as the elevation suitable for agriculture is shifting with climate change and because different elevations are associated with different mean annual temperatures (Tolessa et al., 2017; Ahmed et al., 2019). Eighteen of the articles included in this review examined the effects of cultivating coffee at increased altitude on coffee quality (Figure 3). The majority of articles examining altitude focused on the quality parameter of sensory attributes (*n* = 13) including overall cup quality and taster preference. Twelve articles found that cultivating coffee at increased altitude was associated with an increase in sensory attributes (Decazy et al., 2003; Avelino et al., 2005; Oberthür et al., 2011; Bertrand et al., 2012; Ramos et al., 2016; Silva et al., 2016; Silveira et al., 2016; Bote and Vos, 2017; Gamonal et al., 2017; Malau et al., 2017; Tolessa et al., 2017; Worku et al., 2018). Only one article found a decrease in sensory attributes with increased altitude (Bosselmann et al., 2009).

Four of the articles examining the effects of altitude on sensory attributes demonstrated a positive relationship between altitude and acidity (Avelino et al., 2005; Gamonal et al., 2017; Malau et al., 2017, Worku et al., 2018). One of these studies found a positive relationship between altitude and acidity for coffee cultivated in shade-grown systems (Worku et al., 2018). A follow-up study by the same lead author found no significant difference in acidity with an increase in altitude in coffee grown without shade (Worku et al., 2018). Two articles found a positive relationship between increased altitude and body of the brewed cup (Avelino et al., 2005; Bote and Vos, 2017).

The evidence regarding the effect of cultivating coffee at higher altitudes on metabolites was mixed. Several articles noted an inverse relationship between increased altitude and specific secondary metabolites while other articles noted a direct relationship. For example, three articles found that increased altitude was associated with coffee with lower levels of specific methylxanthines/ alkaloids (Tolessa et al., 2017; Hagos et al., 2018; Worku et al., 2018) while one article found higher levels of these compounds with increased altitude (Avelino et al., 2005). Two articles demonstrated a decrease in phenolic compounds of coffee cultivated at higher altitudes (Tolessa et al., 2017; Worku et al., 2018). Two articles reported lower levels of volatile terpenes with increased altitude (Bertrand et al., 2012; Sridevi and Giridhar, 2016) while one article showed an increase in terpene levels (Rubayiza and Meurens, 2005). Two articles found that coffee grown at increased altitude was associated with increased sugars (Joët et al., 2010; Worku et al., 2018) while one article reported decreased sugar levels in coffee cultivated at increased elevation (Avelino et al., 2005). The one article that assessed responses of lipids to altitude found an increase in lipids with coffee grown at higher altitude (Decazy et al., 2003).

Several of the articles focusing on altitude and coffee quality measured both secondary metabolites as well as sensory attributes and, examined the relationship between these quality parameters. For example, Tolessa et al. (2017) observed a decrease in multiple secondary metabolites including caffeine and chlorogenic acids along with an increase in sensory attributes in high-grown coffee (1,950–2,010 m.a.s.l.) as compared to lower-altitude coffee (1,600–1,680 m.a.s.l.; with a mean annual temperature difference of 3.2 ± 0.7°C between the high and low-altitude locations). The temperature variation based on altitude resulted in delayed ripening, prolonged fruit-fill, and higher accumulation of flavor and aroma precursors (Tolessa et al., 2017). Three additional articles report similar results with regards to improved sensory attributes and reduced secondary metabolites in high-elevation coffee compared to low-elevation coffee (Avelino et al., 2005; Joët et al., 2010; Cruz Bolivar et al., 2017).

### Light Exposure

**Figure 4:**
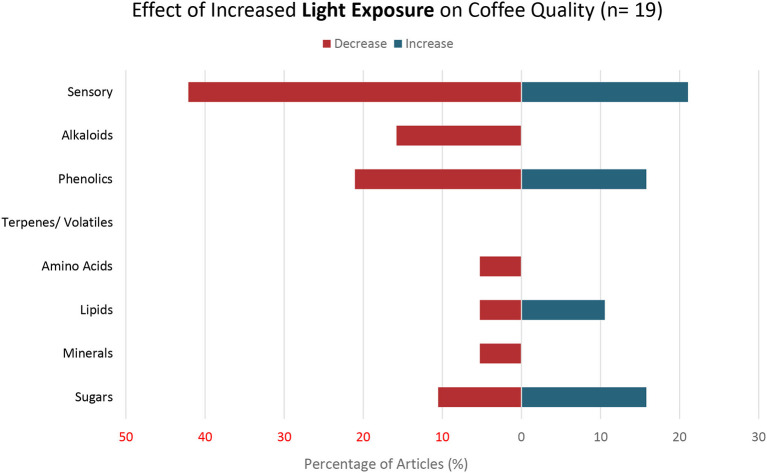
Effects of Increased Light Exposure on Coffee Quality. Percent of articles out a total of 19 articles that reported either an increase, decrease, or both increase and decrease in specific coffee quality parameters with increased light exposure.

Nineteen of the reviewed articles assessed the effects of changes in light exposure on coffee quality (Figure 4). The light exposure examined in the articles focused on either the amount of light in coffee production systems such as solar radiation (directly related to light exposure and inversely related to percentage of shade) or on shade variables that decrease light exposure including canopy density and diversity, percent shade, and planting density (or self-shading).

Sensory attributes were the most prevalent coffee quality parameters (*n* = 12) examined in the articles on light exposure. Eight of the 12 articles examining effects of increased light exposure on sensory attributes of coffee found a decrease in coffee quality (Muschler, 2001; Decazy et al., 2003; Silva et al., 2005, 2016; Läderach et al., 2011; Bote and Vos, 2017; Pereira et al., 2018; Prado et al., 2018). Four articles found an increase in sensory attributes with increased light exposure (Bosselmann et al., 2009; Läderach et al., 2011; Silveira et al., 2016; Tolessa et al., 2017). One article observed decreased shade below 45% was associated with brewed coffee that was bitter, grassy, astringent, and lacking aroma (Decazy et al., 2003). Another study found that an increase in shade from 37 to 61% in coffee production systems resulted in improved body of brewed coffee (Läderach et al., 2011).

Four articles found a decrease in phenolic metabolites with an increase in light exposure (Somporn et al., 2012; Delaroza et al., 2017; Tolessa et al., 2017; dos Santos Scholz et al., 2018) while three other articles demonstrated an increase in phenolics with increasing light exposure (Somporn et al., 2012; Tolessa et al., 2017; dos Santos Scholz et al., 2018). Several articles found both an increase and a decrease of various phenolic metabolites with increasing light exposure (Somporn et al., 2012; Tolessa et al., 2017; dos Santos Scholz et al., 2018). The articles examining the effects of increased light exposure on methylxanthines/alkaloids (*n* = 3) found an inverse relationship (Delaroza et al., 2017; Tolessa et al., 2017; Rakocevic et al., 2018).

Two articles found increased light exposure resulted in increased levels of lipids (Delaroza et al., 2017; Rakocevic et al., 2018) while one article found a decrease in lipids with increased light (dos Santos Scholz et al., 2018). Two articles found an increase in sugar levels with increased light (Somporn et al., 2012; Worku et al., 2018) while two other articles found a decrease in sugar levels with increased light (Somporn et al., 2012; dos Santos Scholz et al., 2018). One article found a decrease in amino acids with increasing light factors (Rakocevic et al., 2018).

One article that examined the interaction of increased light exposure with nutrient management did not find any changes on coffee quality with increased unless coffee plants showed nitrogen (N) deficiency (Bote and Vos, 2017). When light increased and N was limited, acidity, aftertaste, flavor, balance, and overall preference declined (Bote and Vos, 2017). Conversely, two multivariable articles indicated that at higher altitudes, coffee sensory attributes increased with light exposure (Silveira et al., 2016; Tolessa et al., 2017). Another study highlighted that at mid altitude, total chlorogenic acids increased with increasing light exposure while at high altitude total chlorogenic acids decreased with increasing light exposure (Tolessa et al., 2017). Another study found an increase in total chlorogenic acids with increased light as a result of lower planting density (dos Santos Scholz et al., 2018). The response of coffee quality parameters to shifts in light exposure was not always linear. One article revealed a non-linear relationship between chlorogenic acids and light, with the highest levels of chlorogenic acids being highest in coffee grown in natural shade (>70%) followed by coffee grown under artificial shade of 60, 70, 50%, and lastly under full sun (Somporn et al., 2012). Tolessa et al. (2017) and Delaroza et al. (2017) found caffeine content decreased with increased light due to less shade from the canopy.

### Temperature

**Figure 5:**
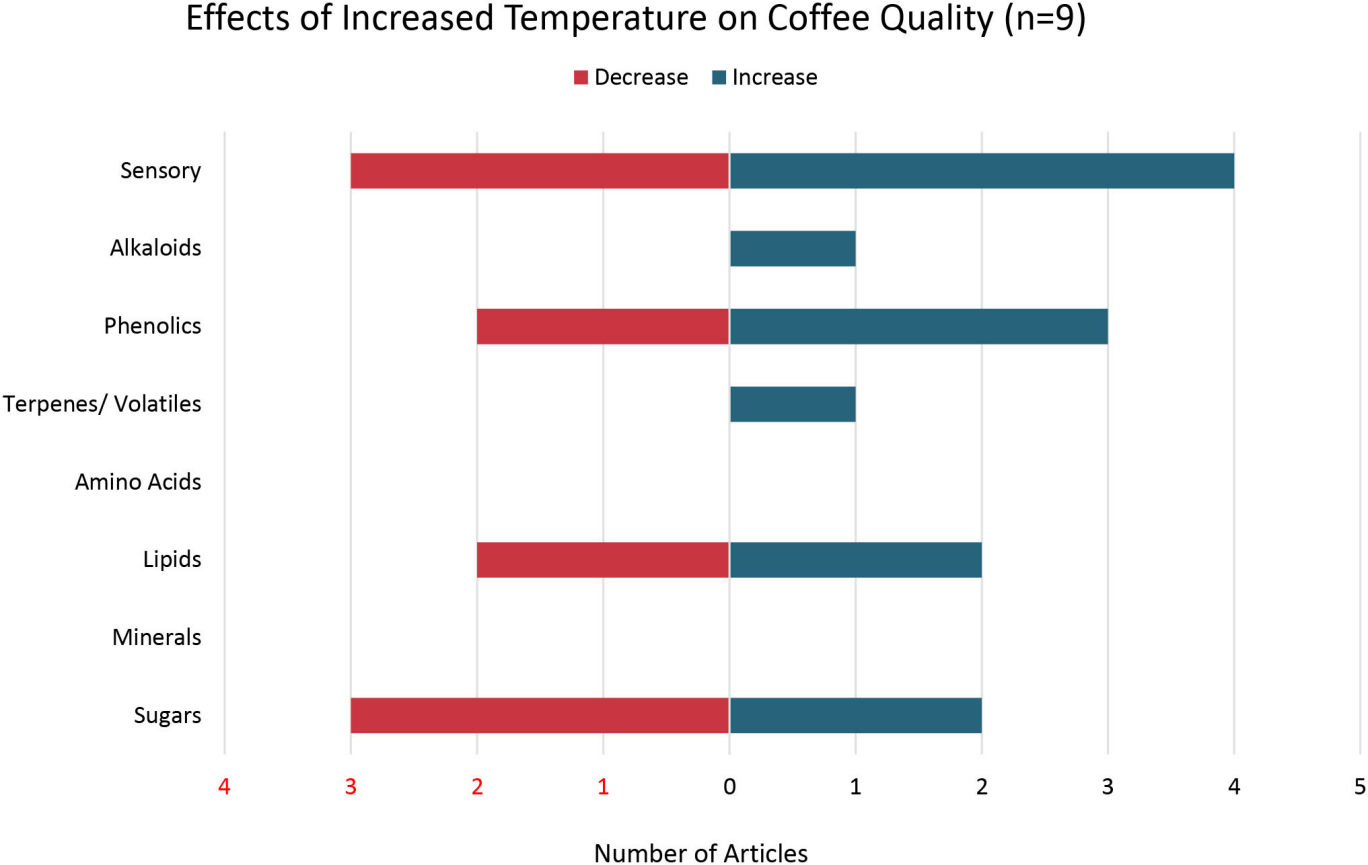
Effects of Increased Temperature on Coffee Quality. Number of articles out of a total of 9 articles that reported either an increase, decrease, or both increase and decrease in specific coffee quality parameters with increased temperature.

Nine out of the 73 articles examined the effects of temperature on coffee quality (Figure 5). Four articles found that coffee grown under increased temperature resulted in an increase in sensory attributes (Silva et al., 2005; Oberthür et al., 2011; de Oliveira Aparecido et al., 2018; Ramalho et al., 2018) while three articles found a decrease in sensory attributes (Silva et al., 2005; Oberthür et al., 2011; Bertrand et al., 2012). For example, Bertrand et al. (2012) reported that warm mean air temperature during seed development of coffee plants was correlated with negative sensory attributes in coffee such as off-flavors and that warmer temperatures overall had an inverse relationship with coffee acidity, fruity flavors, and overall flavor quality.

Two studies found no change in phenolic concentrations with rising temperatures (Joët et al., 2010; Somporn et al., 2012) while one study found an increase in phenolics with increased temperature (Ramalho et al., 2018). For example, Ramalho et al. (2018) found that concentrations of p-coumaric acid, 3-caffeoylquinic acid, 4-caffeoylquinic acid, and total chlorogenic acids as well as methylxanthine metabolites increased with a rise in temperature. Specifically, concentrations of the methylxanthine metabolites caffeine and trigonelline were positively correlated with increased temperatures during the final 4 months of coffee fruit ripening (Ramalho et al., 2018). Mixed results were found for coffee sensory attributes, given that an increase in phenolics and caffeine above specific thresholds is associated with reduced sensory attributes due to perceived bitterness while an increase in trigonelline, a precursor to flavor and aroma compounds, is associated with improved sensory attributes (Barbosa et al., 2012; Ramalho et al., 2018).

The one study that examined the effects of increased temperature on aromatic volatile metabolites found an increase in most volatiles including three volatiles that are associated with reduced sensory attributes because of their green and earthy flavors (2-phenylethanol, butan-1,3-diol and butan-2,3-diol) (Bertrand et al., 2012). Conversely, Bertrand et al. (2012) found a negative correlation between higher temperature and ethanal, a volatile that is associated with the acidity, fruity attributes, and overall quality of coffee (Bertrand et al., 2012).

Of the three studies examining the effects of increased temperature on coffee lipid content, one study found an increase in lipids (Villarreal et al., 2009), one study found a decrease in lipids (Dussert et al., 2001), and one study found both an increase and decrease in various lipids (Joët et al., 2010). For example, Villarreal et al. (2009) found that linoleic and palmitic acids were negatively correlated with increased temperature while and oleic and stearic acids were positively correlated with increased temperature (Villarreal et al., 2009). One article found both an increase and a decrease in sugars such as glucose and stachyose with increased temperature (Joët et al., 2010).

### Water Stress

**Figure 6:**
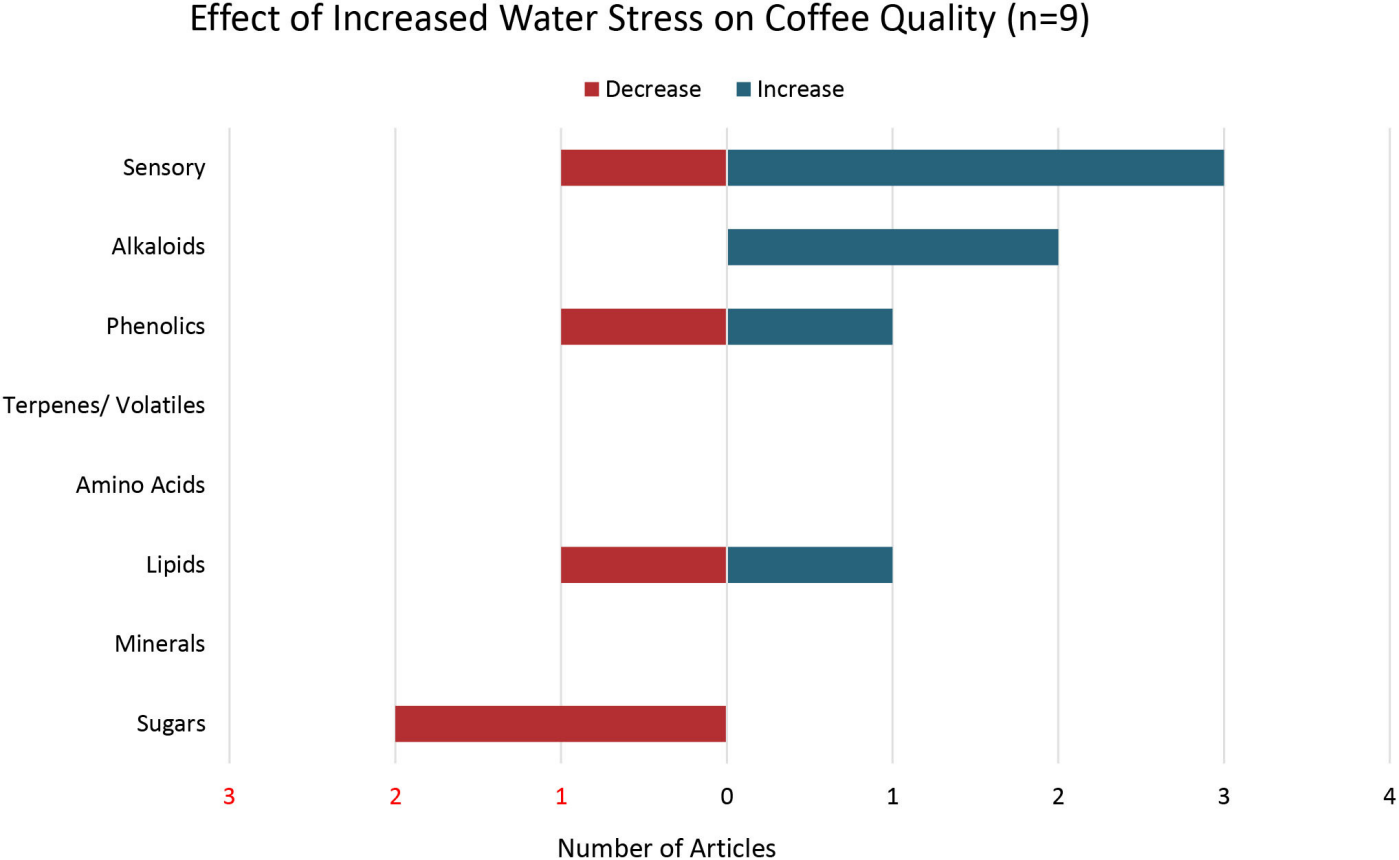
Effects of Increased Water Stress on Coffee Quality. Number of articles out a total of 9 articles that reported either an increase, decrease, or both increase and decrease in specific coffee quality parameters with increased water stress.

The nine articles that examined the effects of water stress on coffee quality (Figure 6) demonstrate mixed evidence. Two articles found increased sensory attributes with increased water stress (Agwanda et al., 2003; Decazy et al., 2003) while one article found both an increase and decrease in coffee quality (Oberthür et al., 2011). For example, Decazy et al. (2003) found that rainfall under 1,600 mm/year was associated with improved sensory attributes of coffee. Agwanda et al. (2003) reported that moderate water stress during the fruit-fill plant developmental stage that occurs 4–6 months after flowering resulted in improved sensory attributes of coffee. Oberthür et al. (2011) noted that the effects of water stress on coffee sensory attributes varied with geographic location. In one region, higher rainfall (above 2,081 mm/ year) enhanced flavor and acidity while in another region, higher rainfall enhanced flavor but not acidity (Oberthür et al., 2011). In the latter region, the number of dry months (rainfall <90 mm) was also critical for flavor development of coffee (Oberthür et al., 2011). Overall, Oberthür et al. (2011) found that steady annual precipitation throughout fruit development and a dry period of 1–3 months leading up to harvest is important for coffee quality.

Two articles found an increase in methylxanthine/alkaloid metabolites with increased water stress (Liu et al., 2016; Vinecky et al., 2017) while one article found a decrease in phenolic levels with increasing water stress (Liu et al., 2016). For example, Liu et al. (2016) found that water deficits of 40 and 60% were associated with an increase in caffeine, while the 40% water deficit was associated with a decrease in chlorogenic acids (Liu et al., 2016). One article found an increase in lipids (Decazy et al., 2003) while another study found a decrease in lipids (Vinecky et al., 2017). Two articles reported a decrease in sugars with increasing water stress (Agwanda et al., 2003; Vinecky et al., 2017). Excess rain was found to negatively impact coffee quality in several studies (Decazy et al., 2003; Oberthür et al., 2011).

### Nutrient Management

**Figure 7:**
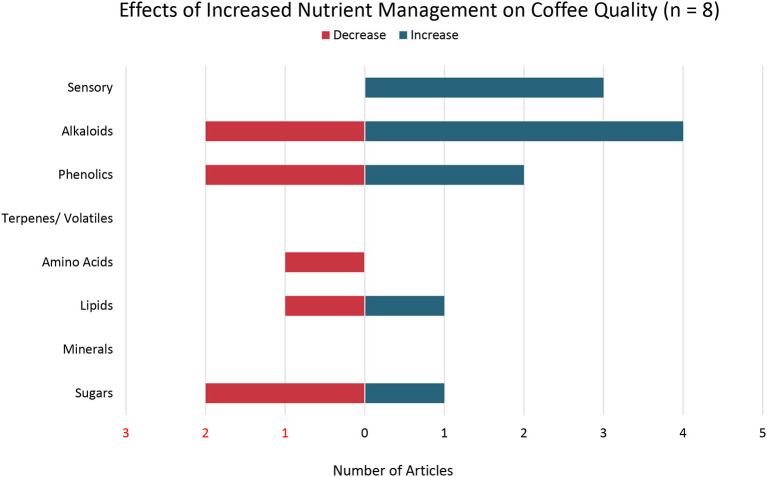
Effects of Increased Nutrient Management on Coffee Quality. Number of articles out a total of 8 articles that reported either an increase, decrease, or both increase and decrease in specific coffee quality parameters with increased nutrient management.

Eight studies in this systematic review examined the effects of nutrient management on coffee quality and found varied results (Figure 7). In most cases, nutrient management was examined along with other environmental factors (such as precipitation, light exposure, and altitude) and management practices in coffee production systems. Several articles found that an increase in macronutrient content of soils was associated with an increase in sensory attributes of coffee (Clemente et al., 2015; Bote and Vos, 2017). For example, Vinecky et al. (2017) found increasing potassium fertilization led to an increase in lipids and chlorogenic acids in coffee while increasing nitrogen application led to increased caffeine content. Clemente et al. (2015) found increased potassium fertilization led to an increase in chlorogenic acids and other phenols in addition to total sugars and caffeine. The relationship between potassium and caffeine, however, was not linear, indicating an optimal dose of potassium at which caffeine is maximized (Clemente et al., 2015). Clemente et al. (2015) further reported that total titratable acidity of coffee decreased with increased potassium application. One study found that reducing overall nitrogen application led to increased chlorogenic acid content in coffee (Liu et al., 2016).

The reviewed studies further pointed to variable responses of coffee to macronutrient application dependent on other environmental and management conditions such as water stress. For example, under moderate irrigation conditions, nitrogen application was associated with a high-quality cup of coffee based on sensory attributes as well as increased protein, crude fat, and chlorogenic acids (Liu et al., 2016). Lower irrigation under the same high nitrogen application rate resulted in increased caffeine content and crude fiber (Liu et al., 2016). Another study found decreased coffee quality when nitrogen supply was limiting under full sunlight conditions, and improved overall coffee bean quality once nitrogen was increased to a non-limiting quantity regardless of light conditions (Bote and Vos, 2017).

Findings in the articles on the effects of nutrient management on coffee secondary metabolites found mixed results. For example, Pech-Kú et al. (2018) found acidic soil with relatively high concentrations of aluminum activates coffee secondary metabolites including caffeine. Application of boron, copper, and zinc *via* foliar spray on coffee plants resulted in increased caffeine, trigonelline, and sucrose levels (Clemente et al., 2018). Addition of zinc to the boron treatment resulted in increased levels of the sugars arabinose and mannose (Clemente et al., 2018). Concentrations of the secondary metabolites 3-O-caffeoylquinic acid (3-CQA) and 5-O-caffeoylquinic acid (5-CQA) were lower when foliar nutrient sprays were applied to coffee plants than without the addition of micronutrients (Clemente et al., 2018).

### Type of Cultivar

Three of the reviewed articles examined coffee cultivar selection as a potential management practice for climate adaptation as well as effects on sensory attributes (Van Der Vossen, 2009) and secondary metabolites (Bertrand et al., 2003; de Oliveira Fassio et al., 2016). One article found enhanced coffee sensory attributes in cultivars derived from Timor Hybrid crosses that are climate-resilient and disease resistant compared to the control cultivars (Van Der Vossen, 2009). For example, de Oliveira Fassio et al. (2016) found variation in levels of the alkaloids, caffeine and trigonelline between cultivars. Bertrand's et al. (2003) examination of Timor Hybrid-derived genetic lines showed variation, in every sugars, lipids, alkaloids, and phenolic metabolites. The planting environment of the production system was demonstrated to modify the effect of cultivar variation and genotype on coffee quality (Bertrand et al., 2003).

### Pest and Disease Management

The two articles examining the impact of pest and disease mitigation on coffee quality found an increase on multiple quality parameters with the exception of acidity (Vaddadi and Parvatam, 2015; Walker et al., 2018). For example, the application of *Rhizopus oligosporus* and *Aspergillus niger* mycelial extracts as well as methyl jasmonate and salicylic acid resulted in increased levels of caffeine up to 42%, increased levels of theobromine up to 39%, increased levels of trigonelline up to 46%, and increased levels cafestol and kahweol up to 32% (Vaddadi and Parvatam, 2015). Walker et al. (2018) found infestation by the coffee berry borer resulted in increased levels of key aromatic volatile metabolites often associated with off-flavors and aromas such as malty, green bean, and grassy attributes including the metabolites hexanal, 2-pentylfuran, 2-methylpropanal, 3-methylbutanal, 2-methylbutanal, benzeneacetaldehyde, and nonanal.

### Fruit Thinning

One article (Bote and Vos, 2017) examined the impacts of manual fruit thinning, a practice to decrease fruit load on coffee quality (higher fruit load is associated with reduced seed size owing to carbohydrate competition among berries during fruit-fill; Vaast et al., 2006). This study found that manual fruit thinning was associated with improved sensory attributes up until 50% thinning at high altitude (1,750–2,100 m.a.s.l.) as compared to mid and low altitudes (1,550–1,750 m and 1,000–1,550 m.a.s.l.); thinning beyond 50% did not improve sensory attributes (Bote and Vos, 2017).

### Carbon Dioxide

Only one paper was identified through the systematic review process on the effects of elevated CO_2_ concentrations on coffee quality (Ramalho et al., 2018). Increased CO_2_ concentration did not have a notable impact on coffee quality based on physical or chemical attributes (Ramalho et al., 2018). However, the interaction of increased CO_2_ and increased temperature acted to mitigate some of the detrimental effects of high temperature on coffee quality including maintaining total chlorogenic acids at a similar level as under optimal temperature conditions (Ramalho et al., 2018).

## Discussion

Given the lack of long-term studies examining the effects of climate change on coffee quality, evidence on the impacts of environmental and management conditions on coffee quality is critical to understand the resilience and vulnerability of the coffee system toward informing future research and evidence-based innovations for climate adaptation. The totality of evidence from the 73 articles identified and reviewed through this systematic review indicate that coffee quality is vulnerable to shifts in environmental and management conditions. Both increases and decreases were found in the sensory attributes and secondary metabolite profiles that determine coffee quality in response to variable environmental and management conditions including changes in water stress, temperature, light exposure, carbon dioxide, and nutrient management. Many studies demonstrated a non-linear response of coffee quality to changes in environmental and management conditions. The greatest evidence base regarding the number of published studies and consistency of evidence was found for altitude and light exposure/shade management. Specifically, the most consistent trends identified through this systematic review include the following: (1) increased altitude was associated with enhanced sensory attributes and; (2) decreased light exposure/increased shade up to certain thresholds was associated with enhanced coffee sensory attributes. Evidence gaps regarding the number of published studies and consistency of evidence was found for the environmental factors of carbon dioxide, temperature, and water stress as well as for the interaction between multiple environmental factors and management conditions. Several management conditions were identified as promising strategies for mitigating the effects of climate change on coffee quality that warrant additional research including shade management, selection of climate-resilient cultivars, tapping into wild coffee germplasm, soil nutrient management, and integrated pest management. Overall, greater evidence was found for the coffee quality outcome of sensory attributes compared to secondary metabolites, with a lack of studies examining the interaction between quality parameters. On the basis of the totality of evidence and the gaps identified, we conclude with recommendations for future research toward the design of evidence-based innovations for strengthening the resilience and sustainability of the coffee system from farm to cup.

The evidence synthesized in this systematic review demonstrates that coffee quality is vulnerable to environmental factors associated with climate change including shifts in light exposure, water stress, temperature, and carbon dioxide. These findings highlight the narrow threshold of coffee with regards to optimum water and temperature thresholds and, are aligned with previous research in other plants such as tea (Ahmed et al., 2014b). Findings on the sensitivity of coffee quality to environmental and management conditions is consistent with other specialty crops (Ahmed and Stepp, 2016). For example, rainfall, temperature, and humidity were found to influence terpenes and volatiles in apples (Vallat et al., 2005). Alpha-tocotrienol and/or alpha-tocopherol in brown rice increased at elevated temperature whereas gamma-tocopherol and gamma-tocotrienol decreased (Britz et al., 2007). Cooler temperatures were found to be positively correlated to phenolic compounds and antioxidant properties in grapes (Xu et al., 2011). Concentrations of alpha-acids, beta-acids, desmethylxantho-umol, and xanthohumol in hops were found to be depended on climatological conditions (Keukeleire et al., 2007).

The greatest evidence base regarding the number of published studies was found for geography (31 articles), altitude (18 articles), and light exposure (19 articles). Altitude and light exposure were also the environmental factors with the most consistent evidence regarding the effects on the directionality (increase, decrease, or non-linear) of coffee quality for sensory attributes. Evidence regarding the effects of altitude and light exposure on secondary metabolites was less consistent compared to sensory attributes; this inconsistent evidence is largely due to the fewer number of studies on secondary metabolites compared to sensory attributes. A moderate evidence base regarding the number of published studies was found for the effects of temperature (9 articles) and water stress (9 articles). Likewise, temperature and water stress had inconsistent evidence regarding effects on the directionality (increase, decrease, or non-linear) of coffee quality. For example, in some cases increased temperature was found to result in improved sensory attributes of coffee (Silva et al., 2005; Oberthür et al., 2011; de Oliveira Aparecido et al., 2018; Ramalho et al., 2018) while in other cases it was found to result in a decrease in sensory attributes (Silva et al., 2005; Oberthür et al., 2011; Bertrand et al., 2012). Likewise, in some cases increased water stress was found to result in increased sensory attributes of coffee (Agwanda et al., 2003; Decazy et al., 2003) while in other cases it was found to result in both an increase and decrease in coffee quality (Oberthür et al., 2011).

The least amount of evidence for environmental factors based on the number of articles was for carbon dioxide (1 article), highlighting a critical area for future research. Understanding the effects of shifts in carbon dioxide is especially important given forecasted trends with climate change coupled with evidence on resulting shifts in crop yields and concentrations of certain primary metabolites (Myers et al., 2014) as well as secondary metabolites (Li et al., 2017) for certain crops. A recent review by Ainsworth and Long (2020) found that elevated carbon dioxide caused an increase in yields in C3 plants under non-stress conditions as well as a reduced of nutrients for many crops. Li et al. (2017) found that elevated carbon dioxide was associated with significant increase in concentrations of total catechins and other polyphenols along with theanine and free amino acids while levels of caffeine decreased. Future research examining the effects of increased carbon dioxide levels on crop quality should draw from lessons learnt from previous free-air carbon dioxide enrichment experiments (Ainsworth and Long, 2020).

In addition to environmental factors, this systematic review found that coffee quality is sensitive to shifts in management conditions. For example, simulated and actual pest infestation was found to increase various coffee quality parameters (Vaddadi and Parvatam, 2015; Walker et al., 2018). This trend is line with previous research on the upregulation of secondary metabolite defense compounds with herbivory (Scott et al., 2020). As climate change impacts crop quality around the world, tolerance of specific herbivores in diversified agroecosystems such as through precision agriculture may be one solution for enhancing crop quality and mitigating the repercussion of climate change such as water stress (Ahmed et al., 2014a). The management condition with the greatest number of articles was nutrient management (8) with a few articles on other management conditions including type of cultivar (3 articles), pests and disease (2 articles), and fruit thinning (1 article). Given the lack of studies on management conditions, no clear trends emerged regarding effects on the directionality of coffee quality.

Interactive effects were found between environmental and management conditions in multiple studies. In some cases, management conditions offset the impacts of environmental variation on coffee quality. In other cases, management conditions only impacted coffee quality dependent on the presence of specific environmental conditions. For example, nitrogen fertilization was shown to improve coffee quality based on sensory attributes in the presence of adequate water availability (Liu et al., 2016).

This systematic review highlights that coffee quality is complex. An increase in the concentration of some secondary metabolites is linked to increased sensory attributes whereas an increase in other secondary metabolites is linked to decreased sensory attributes. In addition, an increase in secondary metabolites up to specific thresholds result in positive sensory attributes; sensory attributes then decrease once these thresholds are crossed.

The two consistent trends identified in this systematic review including increased altitude being associated with improved sensory attributes and increased light exposure associated with decreased sensory attributes (Avelino et al., 2005; Joët et al., 2010; Cruz Bolivar et al., 2017; Tolessa et al., 2017) is aligned to research on other crops including tea (Ahmed et al., 2013, 2019) and cacao (Carrillo et al., 2014). Examining crops cultivated at different altitudes is often used as a proxy for temperature (Kfoury et al., 2019). Higher altitudes are associated with cooler temperatures that result in slower ripening, prolonged fruit-fill, and higher accumulation of flavor (taste and aroma) precursors (Bertrand et al., 2006, 2012; Vaast et al., 2006). Findings of this review coupled with global evidence of an upward shift of plant species to high altitude ecosystems (Pauli et al., 1996; Morueta-Holme et al., 2015; Fadrique et al., 2018) suggest that coffee quality in lower elevation coffee-producing areas is more vulnerable than at higher elevations. The vulnerability of coffee quality at lower elevations provides insight on what may happen to coffee quality at higher elevations in the future with increased temperature linked to climate change, highlighting that climate adaptation is needed for coffee agricultural systems at all elevations.

While the evidence synthesized in this review suggests that relocating coffee to higher elevations can support coffee quality, it is important to recognize the multiple limitations of relocating coffee production systems as well as the opportunities for fostering climate adaptation in vulnerable low-elevation areas. Specifically, relocating coffee to higher elevations is confronted with the following limitations: (1) limited land; (2) potential threats to biodiversity associated with conversion of forested land to agriculture; (3) economic challenges for small-holder farmers to purchase affordable land; (4) socio-cultural challenges associated with moving small-holder communities who may have knowledge and identity tied to a place; (5) infrastructure / resource limitations such as road access and electricity and; (6) future threats to high-elevation areas with climate change. Given these limitations, there is a need to implement climate adaptation innovations to support coffee quality at all elevations, particularly in vulnerable small-holder communities who have a long cultural-history tied to coffee production.

Evidence from this review demonstrates that different coffee producing regions have specific coffee quality profiles that determine a geographic signature of *terroir*. However, no clear trends were found for geographic areas that are more vulnerable or suitable with regards to coffee quality. Given the northward shift of agricultural under twenty-first-century global climate change scenarios (King et al., 2018), future research should evaluate variability of quality across coffee producing locations over time as well as determine the vulnerability of coffee-producing locations at lower latitudes and the suitability of coffee-producing locations at higher latitudes. Similar to relocating farms to higher elevations, moving coffee farms to higher latitudes faces numerous limitations which threaten the livelihood of small-holder coffee producers in the global South. Many small-holder producers have ecological knowledge and cultural history tied to a place (Ahmed et al., 2014b) which would be at risk when uprooting a community with a northward expansion of coffee agriculture.

Climate effects on coffee have notable implications not only for the coffee sector but for society more broadly. For example, the surge in migrants from Guatemala attempting to cross the southern United States border in 2019 was linked to low coffee prices and decreased coffee due to coffee rust (Sieff, 2019; Leutert et al., 2020). Thus, changes occurring on coffee farms not only impact the coffee industry, but society more broadly including international relationships, calling for the urgent need for climate adaptation.

Shade management is a promising and feasible climate-adaptation practice given evidence of the inverse relationship between light exposure and coffee quality found in this systematic review coupled with the benefits of shade-grown coffee for advancing sustainability including biodiversity and ecosystem services (Lin, 2007). While light exposure is shifting with climate change, this variable can be modified in coffee agricultural systems through shade management including fostering shade-grown coffee systems with optimal levels of canopy coverage. Shade management is a relatively accessible climate adaptation strategy compared to alternatives such as relocating farms. Several studies found coffee quality increased under moderate to high canopy cover and shade (45–70% shade) (Decazy et al., 2003; Läderach et al., 2011; Bote and Vos, 2017). Shade-grown coffee systems and other forms of agroforestry are recognized as climate-resilience strategies (Lin, 2007; Ahmed et al., 2014b, 2019) that contribute to multiple dimensions of sustainability including conservation of biodiversity (Jha et al., 2014), sequestering carbon (van Rikxoort et al., 2014), and providing ecosystem services (Lin, 2007). While resource constraints and other barriers exists for the transition of open-sun coffee farms to diversified shade-grown agroforestry models, the limitations are generally less compared to other alternatives such as relocating coffee farms. One of the major limitations of coffee agroforestry is that coffee trees will not bear fruit for several years and other species within the agroecosystem may also take multiple years to materialize benefits (Läderach et al., 2017). Producer incentives are needed to help overcome challenges for transitioning to diversified agroforestry to enhance the sustainability of the coffee system.

Maintenance of diversified coffee germplasm including wild coffee accessions and climate-resilient cultivars is another promising climate adaptation (Bertrand et al., 2003; DaMatta and Ramalho, 2006; Van Der Vossen, 2009; DaMatta et al., 2018). Wild coffee accessions are critical genetic resources for identifying climate-resilient cultivars given their high genetic diversity and variable responses to climate change (Campa et al., 2005; dos Santos Scholz et al., 2016). This systematic review highlights how wild coffee accessions and different cultivars respond variably to various environmental factors. For example, climate-resilient cultivars derived from Timor Hybrid crosses had enhanced coffee sensory attributes compared to the control cultivars (Van Der Vossen, 2009).

### Limitations

Several limitations were encountered in synthesizing data during this systematic review due to the nature of the articles reviewed which limited the ability to quantitatively synthesize data on the effects of environmental and management conditions on the directionality in coffee quality. These limitations include: (1) variation in research design across studies and lack of standardized experimental designs; (2) variation in definitions of coffee quality; (3) variation in approaches to measure coffee quality; (4) lack of studies examining relationships between sensory attributes and secondary metabolites; (5) lack of disclosure in the articles regarding certain variables such as type of coffee cultivar and management conditions of coffee cultivation; (6) the interactive and confounding nature of environmental and management variables that limit the ability to isolate the effects of a single factor on coffee quality; (7) limited long-term studies tracking temporal variation of coffee quality and; (8) coffee quality does not have a linear response to environmental and management variation and many studies do not capture the range of conditions to demonstrate this response.

In addition, while this systematic review focused on coffee quality, our review found a lack of studies on the socio-ecological implications of shifts in coffee quality. Future research should address these limitations in order to strengthen the evidence base on the effects of environmental and management conditions on coffee quality, and implications for society, in the context of climate change.

### Future Research

As weather patterns shift and global temperatures rise, long-term studies are called for in coffee agroecosystems as well as in controlled experimental setups to evaluate the interactive effects of climate change and adaptation innovations on coffee quality as well as yields. Climate projections of rising temperatures are projected to reduce coffee yields, particularly in low altitude regions (Bunn et al., 2015); research is needed to understand the impacts on coffee quality and how this varies with management practices. Research is particularly needed to understand the effects of shifts in carbon dioxide, water stress, and temperature on coffee quality and how this varies with location, elevation, and management conditions. Overall, we recommend the following research actions for advancing the evidence on climate effects on coffee quality for informing the design of programs and policies to strengthen the sustainability and resilience of the coffee system:

1. Standardized research protocols are needed to allow for comparisons between studies. Such protocols should include widely agreed upon international standards of coffee quality based on secondary metabolites and relationships to sensory attributes.
2. Long-term research is needed to capture variability of environmental exposure and effects on coffee quality throughout the year and over time. For example, increased evapotranspiration rates and water stress are predicted in many parts of the world during some seasons (DaMatta et al., 2018, 2019) while increased extreme and variable precipitation events are projected during other seasons (Baca et al., 2014; Shapiro-Garza et al., 2020).
3. Given the northward shift of agriculture with climate change, research on climate change and coffee quality should occur across geographic areas and at different elevations.
4. Research should examine how climate adaptation strategies in coffee agroecosystems as well as various post-harvest technologies can mitigate climate effects on coffee quality. Research is needed to identify optimal management practices for both coffee yields as well as quality, which often have an inverse relationship (Ahmed et al., 2014b).
5. Both on-farm research and controlled experiments are needed to examine the effects of individual and interactive environmental and management effects on coffee quality.

On the basis of this systematic review, the following research questions on climate change and coffee quality should be prioritized by future research toward informing evidence-based programs and policies for the sustainability of the coffee system:

1. What are the optimal ranges and thresholds for various environmental factors (including light exposure, water stress, temperature, and carbon dioxide) for coffee quality and, how does this vary with location, elevation, and management practices?
2. What are the linkages between the coffee quality parameters of primary and secondary metabolite profiles and sensory attributes?
3. What innovations from farm to cup support coffee quality, yields, sustainability, and the overall resilience of the coffee system?
4. What are barriers and opportunities for climate-adaptation strategies for coffee systems such as diversified shade-grown systems and relocating coffee farms to higher elevations and higher latitudes? Do coffee producers have the willingness and capacity to adapt to climate change including access to natural and social capital such as economic resources, land access, ecological knowledge, and social networks?
5. How do responses of coffee quality to various environmental and management conditions compare to other crops?

### Conclusion

As a culturally-relevant and highly flavorful drink consumed by a large global population, shifts in coffee quality have implications throughout the coffee system, from the smallholder coffee-producing communities in the global South to the 2.6 billion coffee consumers. This systematic review highlights that coffee quality is sensitive to shifts in environmental and management variables linked to climate change and climate adaptation. Synthesis of the totality of evidence across studies points to consistent trends regarding the effects of high altitude and low light exposure on enhanced coffee sensory attributes. Critical research gaps were further found where fewer studies exist including effects of carbon dioxide, temperature, and water stress on the directionality of coffee quality (increase, decrease, or non-linear) as well as the interaction of multiple environmental and management conditions. Research gaps were also found with regards to the coffee quality outcome of secondary metabolites compared with sensory attributes, as well as their relationship. Management conditions such as shade management through agroforestry systems, cultivation of diverse climate-resilient cultivars, integrated pest management, and soil nutrient management were identified as promising strategies to offset negative environmental effects on coffee quality. Given the sensitivity of coffee quality to environmental variation, evidence-based innovations are called for to enhance the sustainability and resilience of the coffee sector in the context of global change. Such efforts should take a participatory research approach with the inclusion of diverse coffee stakeholders farm to cup as well as those that interact and support the coffee sector including practitioners, researchers, and policymakers.

## Data Availability Statement

The original contributions presented in the study are included in the article/Supplementary Materials, further inquiries can be directed to the corresponding author.

## Author Contributions

SA conceived of the systematic review and led the study protocol with input from all authors. SA and SC guided the systematic review teams. ES and SB led the review panel on environmental effects on coffee quality. ES, SB, TW, NV, and EA served on the review panel that identified, extracted, synthesized, and reported findings on the environmental effects on coffee quality. SB, AS, MT, CT, and SC served on the review panel that identified, extracted, synthesized, and reported findings on management effects on coffee quality. SB and ES created the figures. SA, ES, and SB led the writing of the manuscript with contributions from all authors. SA led the revisions of the manuscript for publication. All authors listed have made a substantial, direct and intellectual contribution to the work, and approved it for publication.

## Funding

The authors received funding support for the study presented here from the following agencies: (1) United States National Science Foundation - Award NSF RII Track-2 FEC 1632810 (SA), (2) Tufts University, Tufts Collaborates Award 2019-N302201 (SC), (3) United States National Institute of General Medical Sciences of the National Institutes of Health–Awards P20GM103473 (SA), and Award 5P20GM104417 (SA).

## Author Disclaimer

The content is solely the responsibility of the authors and does not necessarily represent the official views of the National Science Foundation or National Institutes of Health.

## Conflict of Interest

The authors declare that the research was conducted in the absence of any commercial or financial relationships that could be construed as a potential conflict of interest.

## Publisher's Note

All claims expressed in this article are solely those of the authors and do not necessarily represent those of their affiliated organizations, or those of the publisher, the editors and the reviewers. Any product that may be evaluated in this article, or claim that may be made by its manufacturer, is not guaranteed or endorsed by the publisher.
