# Supplementary material for: Climate Change and Coffee Quality: Systematic Review on the Effects of Environmental and Management Variation on Secondary Metabolites and Sensory Attributes of Coffea arabica and Coffea canephora
Source: Front Plant Sci. 2021 Oct 8;12:708013. doi: 10.3389/fpls.2021.708013 (PMC8531415; doi:10.3389/fpls.2021.708013)
Supplement: Supplementary file 1 [file Table_1.docx]

| **"COFFEE"** | **AND** | **"CLIMATE CHANGE"** | **AND** | **"QUALITY"** |
| --- | --- | --- | --- | --- |
| **OR** |  | **OR** |  | **OR** |
| “coffea arabica” *or* coffea* *or* “coffee bean” *or* “coffee berry” |  | “global warming” *or* CO2 *or* “carbon dioxide” *or* “greenhouse gas” *or* season* *or* climate *or* climatic *or* spring *or* summer *or* fall *or* winter *or* “length of season” *or* seasonality *or* monsoon *or* weather *or* “dry season” *or* harvest* *or* “solar radiation” *or* UV *or* ultraviolet *or* irradiation *or* “length *or* daylight” *or* “day length” *or* “sunshine hours” *or* shade *or* shading *or* “light intensity” *or* precipitation *or* rainfall *or* drought *or* flood *or* “water logging” *or* saturation *or* “water stress” *or* dry* *or* geograph* *or* “geographic area” *or* *or*igin *or* latitude *or* slope *or* aspect *or* altitude *or* terroir *or* elevation *or* temperature *or* frost *or* freez* *or* “chilling hour*” *or* wind *or* “soil moisture content” *or* environmental* *or* “environmental variation” *or* “environmental stress” *or* “environmental impact” |  | caffeine *or* phytochemical* *or* phyto-chemical *or* “secondary metabolite” *or* nutrient* *or* methylxanthine *or* alkaloid* *or* polyphenol* *or* antioxidant* *or* lignan* *or* quinide* *or* flavonoid* *or* phenol* *or* ester* *or* terpen* *or* aldehyde* *or* magnesium *or* “phenolic acid*” *or* “chl*or*ogenic acid” *or* “caffeoylquinic acid” *or* “hydrocinnamic acid” *or* volatile* *or* terpenoid* *or* diterpene *or* “carbonyl compound” *or* benzenoid* *or* aroma *or* aromatic *or* “heterocyclic compound” *or* trigonelline* *or* “amino acid” *or* lipid* *or* flav*or* *or* flavour *or* taste *or* aroma *or* sens*or*y *or* *or*ganoleptic *or* chemical* *or* compound* *or* fat* *or* content *or* composition *or* liqu*or* *or* bitter* *or* carbohydrate* *or* od*or*ant* *or* glucose *or* fructose *or* xylose *or* robustness *or* body *or* acidity *or* balance *or* fragrance *or* unif*or*mity *or* intensity *or* sweetness *or* aftertaste *or* viscosity |

**Supplementary Table 1.** Search Terms Addressing the Study Question: What are effects of variation in environmental factors related to climate change on coffee quality?
