# Supplementary material for: Climate Change and Coffee Quality: Systematic Review on the Effects of Environmental and Management Variation on Secondary Metabolites and Sensory Attributes of Coffea arabica and Coffea canephora
Source: Front Plant Sci. 2021 Oct 8;12:708013. doi: 10.3389/fpls.2021.708013 (PMC8531415; doi:10.3389/fpls.2021.708013)
Supplement: Supplementary file 2 [file Table_2.docx]

| **"COFFEE"** | **AND** | **"MANAGEMENT"** | **AND** | **"QUALITY"** |
| --- | --- | --- | --- | --- |
| **OR** |  | **OR** |  | **OR** |
| “coffea arabica” *or* coffea* *or* “coffee bean” *or* “coffee berry” |  | soil *or* “soil acidity” *or* “soil pH” *or* “total P” *or* “total P” *or* “available P” *or* “total N” *or* herbivory *or* pests *or* beetle *or* leafminer *or* moth *or* pathogens *or* pests *or* disease *or* microbes |  | quality *or* caffeine *or* phytochemical* *or* phyto-chemical *or* “secondary metabolite” *or* nutrient* *or* methylxanthine *or* alkaloid* *or* polyphenol* *or* antioxidant* *or* lignan* *or* quinide* *or* flavonoid* *or* phenol* *or* ester* *or* terpen* *or* aldehyde* *or* magnesium *or* “phenolic acid*” *or* “chlorogenic acid” *or* “caffeoylquinic acid” *or* “hydrocinnamic acid” *or* volatile* *or* terpenoid* *or* diterpene *or* “carbonyl compound” *or* benzenoid* *or* aroma *or* aromatic *or* “heterocyclic compound” *or* trigonelline* *or* “amino acid” *or* lipid* *or* flavor *or* flavour *or* taste *or* aroma *or* sensory *or* organoleptic *or* chemical* *or* compound* *or* fat* *or* content *or* composition *or* liquor *or* bitter* *or* carbohydrate* *or* odorant* *or* glucose *or* fructose *or* xylose *or* damascenone *or* furfurylthiol *or* methanethiol *or* methylbutanal *or* methylpropanal *or* robustness |

**Supplementary Table 2.** Search Terms Addressing the Study Question: What are the effects of management practices related to climate change adaptation on coffee quality?
