# Supplementary material for: Climate Change and Coffee Quality: Systematic Review on the Effects of Environmental and Management Variation on Secondary Metabolites and Sensory Attributes of Coffea arabica and Coffea canephora
Source: Front Plant Sci. 2021 Oct 8;12:708013. doi: 10.3389/fpls.2021.708013 (PMC8531415; doi:10.3389/fpls.2021.708013)
Supplement: Supplementary file 3 [file Table_3.docx]

| **Directional change** | **Specific coffee quality parameter** | **Exposure to environmental or management parameter** |
| --- | --- | --- |
| increase, decrease, or no change | phenolic compounds | increase / decrease [environmental/ management factors] |
| increase, decrease, or no change | methylxanthines | increase / decrease [environmental/management factor] |
| increase, decrease, or no change | terpenes or volatiles | increase / decrease [environmental/management factor] |
| increase, decrease, or no change | amino acids | increase / decrease [environmental/management factor] |
| increase, decrease, or no change | sugars | increase / decrease [environmental/management factor] |
| increase, decrease, or no change | lipids | increase / decrease [environmental/management factor] |
| increase, decrease, or no change | minerals | increase / decrease [environmental/management factor] |
| increase, decrease, or no change | sensory qualities | Increase / decrease [environmental/management factor] |

**Supplementary Table 3.** Categorization of specific coffee quality outcomes.
